# Mobility Trends Reports Revealed “Self-isolation Fatigue” in Japan: Use of Mobility Data for Coronavirus Disease Control

**DOI:** 10.31662/jmaj.2020-0031

**Published:** 2020-07-08

**Authors:** Shiho Amagasa, Hiroyuki Kojin, Shigeru Inoue

**Affiliations:** 1 Department of Preventive Medicine and Public Health, Tokyo Medical University, Tokyo, Japan; 2 Department of Quality and Safety Management, Graduate Faculty of Interdisciplinary Research, Faculty of Medicine, University of Yamanashi, Yamanashi, Japan

**Keywords:** mobility, infectious disease, social distancing, transport, epidemiology, public health

The novel coronavirus disease (COVID-19) pandemic is spreading worldwide ^(1)^. In Japan, the number of cases is also increasing rapidly, and since the national and local government declared a state of emergency, the campaign for social distancing and self-isolation has intensified ^(2)^. When considering countermeasures, it is important to understand how mobility among people has actually changed and predict how it will change during infectious disease epidemics. On April 15, Apple Inc. began publishing Mobility Trends Reports to support efforts to prevent the spread of COVID-19 ^(3)^. These mobility data are updated daily based on route search information on Apple Maps, dating back to January 13, 2020, when the first case of an infected person outside of China was confirmed. Data on walking, driving, and mass transit can be checked in 63 countries worldwide and in major cities in those countries. In Japan, the cities included are Tokyo, Nagoya, Osaka, and Fukuoka ^(3)^.

The mobility and number of new COVID-19 cases in Japan since February 18 are shown in Figure 1 ^(4)^. In January and February, mobility on weekends increased consistently, in which it reached its highest level during the 3 day weekend (February 22-24). With January 13 as a baseline, on February 22, driving increased by 62%, mass transit by 47%, and walking by 64%. Thereafter, on February 25, the Ministry of Health, Labour and Welfare announced its Basic Policies for Novel Coronavirus Disease Control accompanied by a call for large-scale events to be canceled. This led to relative decreases in mobility on the first and second weekends in March. However, on the third weekend (March 20-22), that is, 3 day weekend, all at once, the number of people on the move surged: a manifestation of “self-isolation fatigue” or “coronavirus fatigue.” Particularly, the annual announcement of the opening of the cherry blossoms was made in Tokyo on March 14, and that the best time to view the blossoms was during the March 20-22. According to an analysis using Community Mobility Reports from Google ^(5)^, there was a sudden increase in Park visits, particularly in Tokyo, on the 3 day weekend starting on March 20^th^ when the blossoms were at their peak. The number of people has increased by 40% compared with the baseline ^(5)^. Given the incubation period for COVID-19, it is almost definitely the case that the spread of infection was prompted by people going out on the 3 day weekend.

**Figure 1.**
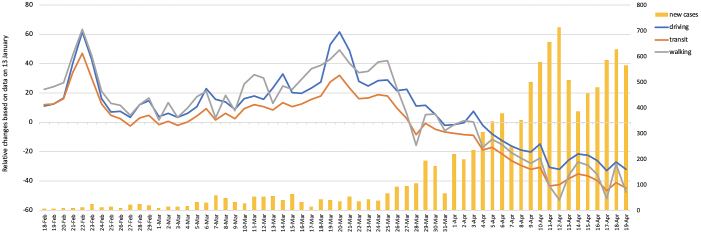
Trends in mobility and numbers of new COVID-19 infections in Japan since February 18, 2020. Trends in mobility and numbers of new COVID-19 infections in Japan are based on Apple’s Mobility Trends Reports ^(3)^ and the novel coronavirus infectious cases report ^(4)^, respectively.

The number of people driving is worth noting. Besides the sudden increase in travel by car on the 3 day weekend in March, many people continued to drive afterward. Although some decrease was evident, that decrease was small compared with the decreases in people moving through mass transit and walking. Automobiles are convenient and afford a high level of freedom and, unlike mass transit, allow people to move about while avoiding close contact with an unspecified large number of people. In general, it is therefore understandable why travel via cars tends not to decrease even during infectious disease epidemics. Given these mobility data, we may anticipate the appearance of coronavirus fatigue and large number of people going out with family or friends via cars during Golden Week (GW; a week-long period with several national holidays covering parts of the last week of April and the first week of May). The worst-case scenario, in which people from urban areas where infections are prevalent travel outside the city and spread the disease to people in provincial areas, is possible to occur.

As of April 19, in Japan, driving had decreased by 32%, mass transit by 45%, and walking by 47% ^(2)^. However, these decreases are smaller than those in the leading industrialized nations where infections are spreading and mobility remains high ^(3)^. This is probably because although a state of emergency has been declared in Japan, it is not backed by a strong legal force. On April 16, the government expanded the state of emergency to the entire nation ^(2)^, one of the aims of which was to minimize the movement of people during GW, but more specific recommendations are needed. Even though a state of emergency has been declared, unless more in-depth measures are taken, it is possible to see an increase in the movement of people during GW from coronavirus fatigue and self-isolation fatigue. Measures to limit driving as well as the use of mass transit may be needed.

## Article Information

### Conflicts of Interest

None

### Approval by Institutional Review Board (IRB)

N/A

### Author Contributions

SA prepared the first draft. HK and SI revised the manuscript. All authors have read and approved the final version of the manuscript.
